# Improved Anticancer Effect of Magnetite Nanocomposite Formulation of GALLIC Acid (Fe_3_O_4_-PEG-GA) Against Lung, Breast and Colon Cancer Cells

**DOI:** 10.3390/nano8020083

**Published:** 2018-02-02

**Authors:** Raihana Rosman, Bullo Saifullah, Sandra Maniam, Dena Dorniani, Mohd Zobir Hussein, Sharida Fakurazi

**Affiliations:** 1 Institute of Biosciences, Universiti Putra Malaysia (UPM), Serdang 43400, Selangor, Malaysia; raihanarosman89@gmail.com (R.R.); bullosaif1@gmail.com (B.S.); 2 Institute of Advanced Technology (ITMA), Universiti Putra Malaysia (UPM), Serdang 43400, Selangor, Malaysia; mzobir@upm.edu.my; 3 Department of Human Anatomy, Faculty of Medicine and Health Sciences, Universiti Putra Malaysia (UPM), Serdang 43400, Selangor, Malaysia; sandra@upm.edu.my; 4 Department of Chemistry, University of Sheffield, Dainton Building, Brook Hill, Sheffield S3 7HF, UK; d.dorniani@sheffield.ac.uk

**Keywords:** gallic acid, magnetite nanoparticles, anticancer, PEG, lung cancer, colon cancer

## Abstract

Lung cancer, breast cancer and colorectal cancer are the most prevalent fatal types of cancers globally. Gallic acid (3,4,5-trihydroxybenzoic acid) is a bioactive compound found in plants and foods, such as white tea, witch hazel and it has been reported to possess anticancer, antioxidant and anti-inflammatory properties. In this study we have redesigned our previously reported anticancer nanocomposite formulation with improved drug loading based on iron oxide magnetite nanoparticles coated with polyethylene glycol and loaded with anticancer drug gallic acid (Fe_3_O_4_-PEG-GA). The in vitro release profile and percentage drug loading were found to be better than our previously reported formulation. The anticancer activity of pure gallic acid (GA), empty carrier (Fe_3_O_4_-PEG) nanocarrier and of anticancer nanocomposite (Fe_3_O_4_-PEG-GA) were screened against human lung cancer cells (A549), human breast cancer cells (MCF-7), human colon cancer cells (HT-29) and normal fibroblast cells (3T3) after incubation of 24, 48 and 72 h using (3-(4,5-dimethylthiazol-2-yl)-2,5-diphenyltetrazolium bromide) MTT assay. The designed formulation (Fe_3_O_4_-PEG-GA) showed better anticancer activity than free gallic acid (GA). The results of the in vitro studies are highly encouraging to conduct the in vivo studies.

## 1. Introduction

Cancer is one of the serious concerns around the world and it is one of the main causes of death worldwide [1]. In a press release by the World Health Organization in 2014, it was reported that cancer accounted for 8.2 million deaths worldwide in 2012 with lung, breast and colorectal cancers identified as most common occurrences worldwide in the year 2012 [2]. This was further supported by a press release by the World Health Organization in 2015. According to the Cancer statistics 2017, in the United States alone, about 1,688,780 new cancer cases and 600,920 cancer deaths are projected to occur [3]. Current cancer treatments rely on radiation and chemotherapeutic agents that work by killing rapidly dividing cells in the body. The main drawback of conventional chemotherapy is the adverse effects on the body as it cannot deliver selective action specifically to the cancer cells, thus damaging the surrounding normal healthy cells or rapidly dividing healthy cells such as the cells of gastrointestinal tract, bone marrow, hair follicles, causing issues like cardiac, hepatic, pulmonary, renal and gastrointestinal toxicities [4,5,6]. Drug delivery systems offer numerous advantages over tradition chemotherapy such as targeted delivery at disease site, sustained release leading to prolonged bioavailability, lower dosage requirement and improved drug solubility among others [7,8,9,10,11,12]. Significant impact has been made by the application of nanotechnology in medicine for theranostic agents development which can diagnose and cure the diseases simultaneously [13,14]. Variety of nanocarriers have been designed and successfully applied for the delivery of the therapeutic agents such as graphene oxide, polymers-based delivery systems, layered double hydroxides, gold nanoparticles, multifunctional nanoparticles and iron oxide magnetite nanoparticles [8,15,16,17,18,19,20,21]. Polymer coated iron oxide magnetite nanoparticles (Fe_3_O_4_) have received much of the attention as the novel cancer therapeutics vectors because of their unique properties such as ease of preparation, easily scalable production, sustained release properties, high encapsulation capacity, biocompatibility with normal cells and tissues, easier surface modification and stable magnetic nature [9,22,23,24,25,26]. All of these characteristics make iron oxide magnetite nanoparticles (Fe_3_O_4_) an ideal candidate for cancer delivery vectors. However, the large surface area to volume ratio and the dipole-dipole attraction causes the agglomeration of nanoparticles, hence the need for surface polymer modification. The magnetic nanocarriers need to be stable in normal saline and water at neutral pH for biological, medical diagnostic and therapeutic applications [23,27]. To avoid agglomeration, the surface of iron oxide magnetite nanoparticles is coated with polymer which also helps in sustained release and better stability in physiological conditions. The polymer poly (ethylene-glycol) (PEG) has been widely applied in drug delivery and is being utilized as protective layer for the nanoparticles. The monomer unit of PEG contains both polar oxygen and two methylene group which are non-polar. This dual polarity makes PEG to be soluble in variety of polar and non-polar solvents and has been widely used to improve aqueous solubility of hydrophobic drugs [28,29]. Gallic acid (3,4,5-trihydroxybenzoic acid) is a bioactive compound found in plants and foods such as white tea, witch hazel and it has been reported to possess antioxidant, anti-inflammatory, anticancer properties and is also known for its protective activity on normal cells which makes them pivotal for cancer therapy [30]. In this study we have redesigned an anticancer nanocomposite formulation of Gallic acid loaded on iron oxide magnetite nanoparticles coated with polyethylene glycol (Fe_3_O_4_-PEG-GA) with improved drug loading and better sustained release properties and was characterized by X-ray diffraction (XRD), dynamic light scattering (DLS), in vitro cytotoxicity assay and drug loading quantification. In previous study we tested Gallic acid nanocomposite formulation [(P-Fe_3_O_4_-PEG-GA) (in formula P stands for previous)] against MCF-7, a breast cancer cell line with the IC_50_ value of 11.61 ± 0.12 µg/mL and human normal lung fibroblast cells MRC-5 was used as a model for normal cell in which more than 80% cell viability was observed after 72 h incubation [31]. In this study we tested the free drug GA, empty nanocarrier and the anticancer nanocomposite formulation Fe_3_O_4_-PEG-GA against A549 human lung carcinoma cells, *HT29* human colon adenocarcinoma cell line, repeated on MCF-7 breast cancer cells and normal 3T3 cells for incubation period of 24, 48 and 72 h.

## 2. Results

### 2.1. Physicochemical Characterization

#### 2.1.1. X-ray Diffraction (XRD) Analysis

**Figure 1:**
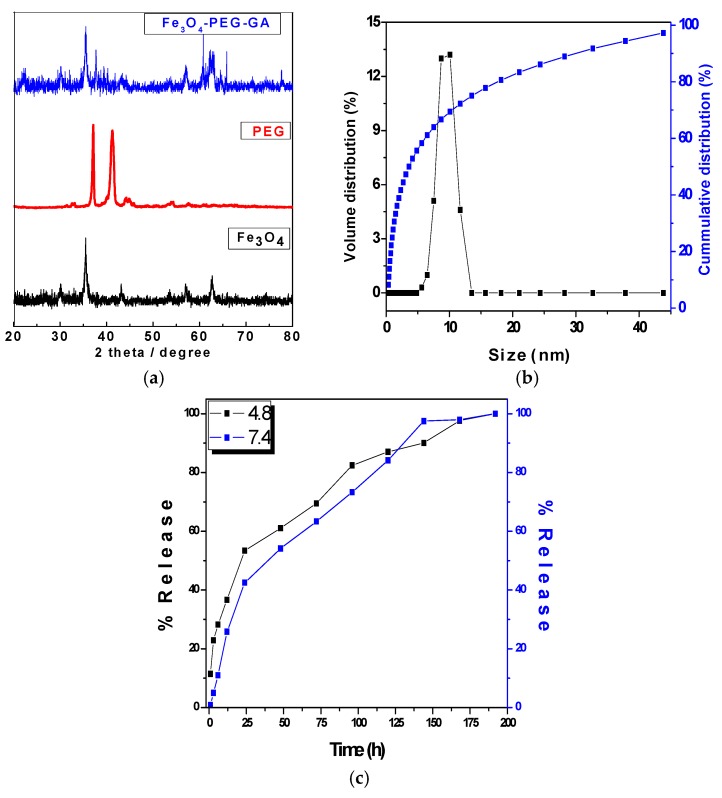
(**a**) X-ray Diffraction (XRD) analysis of iron oxide magnetite nanoparticles (Fe_3_O_4_), poly ethylene glycol (PEG) and anticancer nanocomposite Fe_3_O_4_-PEG-GA; (**b**) Particle size with cumulative and volume distribution of nanocomposite Fe_3_O_4_-PEG-GA; (**c**) Release of GA from the nanocomposite (Fe_3_O_4_-PEG-GA) of iron oxide magnetite nanoparticles (Fe_3_O_4_) coated with polyethylene glycol (PEG) with loaded with gallic acid (GA) being the active anticancer agent.

Figure 1a shows the XRD patterns of iron oxide magnetite nanoparticles (Fe_3_O_4_) alone, poly ethylene glycol (PEG) and anticancer nanocomposite Fe_3_O_4_-PEG-GA. Iron oxide magnetite nanoparticles (Fe_3_O_4_) showed the six characteristics peaks ascribed to Brag reflections due to (220), (311), (400), (422), (511), and (440) and these peaks can be observed at 2*θ* = 30.16°, 35.95°, 43.34°, 54.17°, 57.27° and 62.98° respectively. The pure polymer PEG showed two main characteristics high intensity peaks at about 2*θ* = 19.3° and 23.5°. The pure gallic acid (GA) has been reported to show many peaks between the 2*θ* of 10–50° as reported previously [31,32]. In the XRD patterns of the nanocomposite Fe_3_O_4_-PEG-GA characteristic peaks of iron oxide magnetite nanoparticles (Fe_3_O_4_), PEG and of the pure drug peaks are present with slight lesser intensity. The presence of characteristic peaks of Fe_3_O_4_, PEG and GA in the final anticancer nanocomposite confirms the successful formation of the nanocomposite Fe_3_O_4_-PEG-GA.

#### 2.1.2. Dynamic Light Scattering (DLS)

The size of the anticancer nanocomposite Fe_3_O_4_-PEG-GA was determined using Zetasizer by dynamic light scattering (DLS). The sample was dispersed in water and sonicated for 15 min and then analyzed with Zetasizer. The sample was found to have narrow size distribution between 5 and 12 nm with average particle size of 10 nm as shown in Figure 1b. This size distribution is much smaller compared to previously reported (P-Fe_3_O_4_-PEG-GA) which had wide distribution 20–50 nm with average size of 31.44 nm [31].

#### 2.1.3. In vitro Release Studies

Release behavior of GA from the nanocomposite Fe_3_O_4_-PEG-GA was conducted in human body simulated buffer saline (PBS) solution of pH 7.4 (human blood pH) and in pH 4.8 (intracellular lysosomal pH) as shown in Figure 1c. For the release studies 10 mg of the nanocomposite was put in 10 mL PBS solution of pH 7.4 and pH 4.8 in thermostat at 37 °C with constant shaking. At different time points 3 mL aliquot was taken out and replaced with new buffer of either solution of pH 7.4 and pH 4.8 respectively and analyzed for the percentage release using UV-Vis spectrophotometer (Waltham, MA, USA). The release of GA was found to be sustained in both physiological pHs (7.4 and 4.8) and took 200 h (about 8 days) for the complete release in both conditions as shown in Figure 1c. The release profile of GA from the nanocomposite Fe_3_O_4_-PEG-GA is much more sustained compared to previously designed nanocomposite P-Fe_3_O_4_-PEG-GA which initially showed burst release with more than 40% drug released in less than 2 h [31]. Moreover, the release profile of free drug GA has been reported to be extremely fast which took less than 2 min for the complete release [31]. This suggests that nanocomposite designed (Fe_3_O_4_-PEG-GA) has much better sustain release profile compared to free drug and the previously designed nanocomposite (P-Fe_3_O_4_-PEG-GA).

#### 2.1.4. Drug Loading Percentage Quantification

Percentage loading of the GA in the nanocomposite Fe_3_O_4_-PEG-GA was determined with (UV-Vis) spectrophotomer (Waltham, MA, USA) GA acid was extracted (deloaded) from the nanocomposite Fe_3_O_4_-PEG-GA by putting 10 mg of it in 50 mL of 1 molar phosphate buffer saline (PBS) solution, followed by sonication for an hour and kept in thermostat with constant shaking at 37 °C for 10 days. After that sample was filtered and filtrate was subjected to quantification of GA. For quantification different concentrations of GA standards were prepared e.g., 25, 50, 100, 150 and 200 ppm and were analyzed and correlation coefficient (*r*^2^) was found to be 0.9918. After that different filtrate was analyzed three times and loading was found to be 35%. The percentage loading of GA in this redesigned nanocomposite is much higher (35%) compared to our previously reported designed nanocomposite of Fe_3_O_4_-PEG-GA in which loading percentage of GA was found to be very lower i.e., 7%. The improved loading can be ascribed to solvent used to dissolve GA i.e., (80% Methanol:20% water).

#### 2.1.5. Cytotoxicity on 3T3 Fibroblast Cells

**Figure 2:**
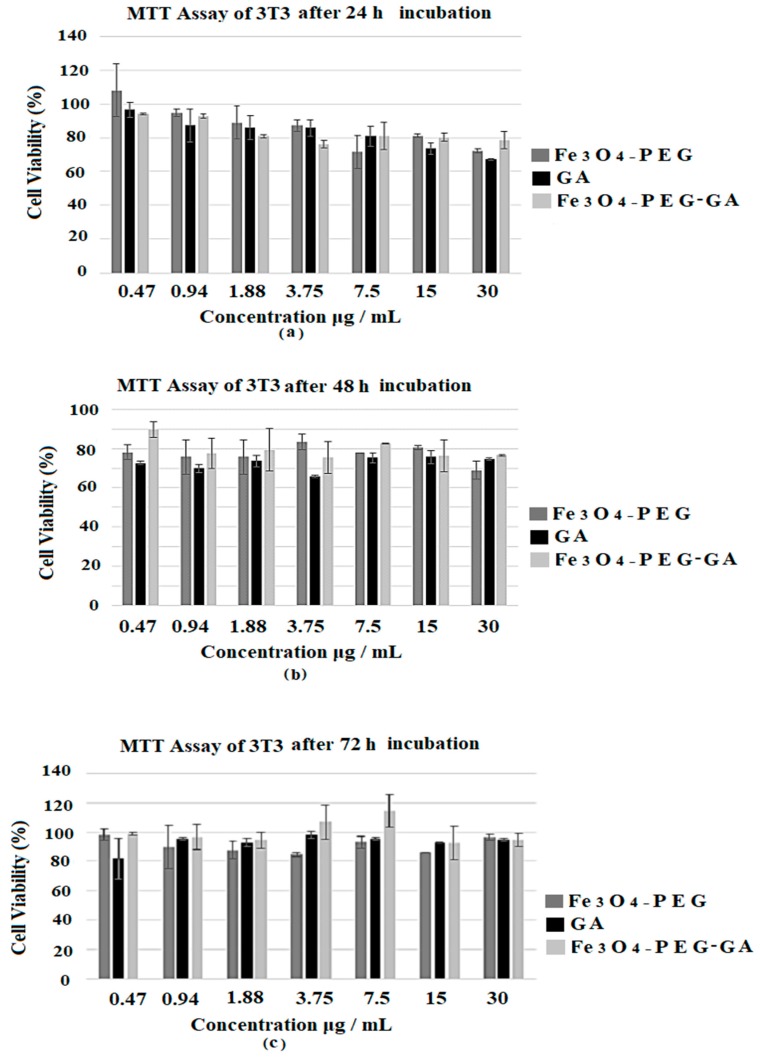

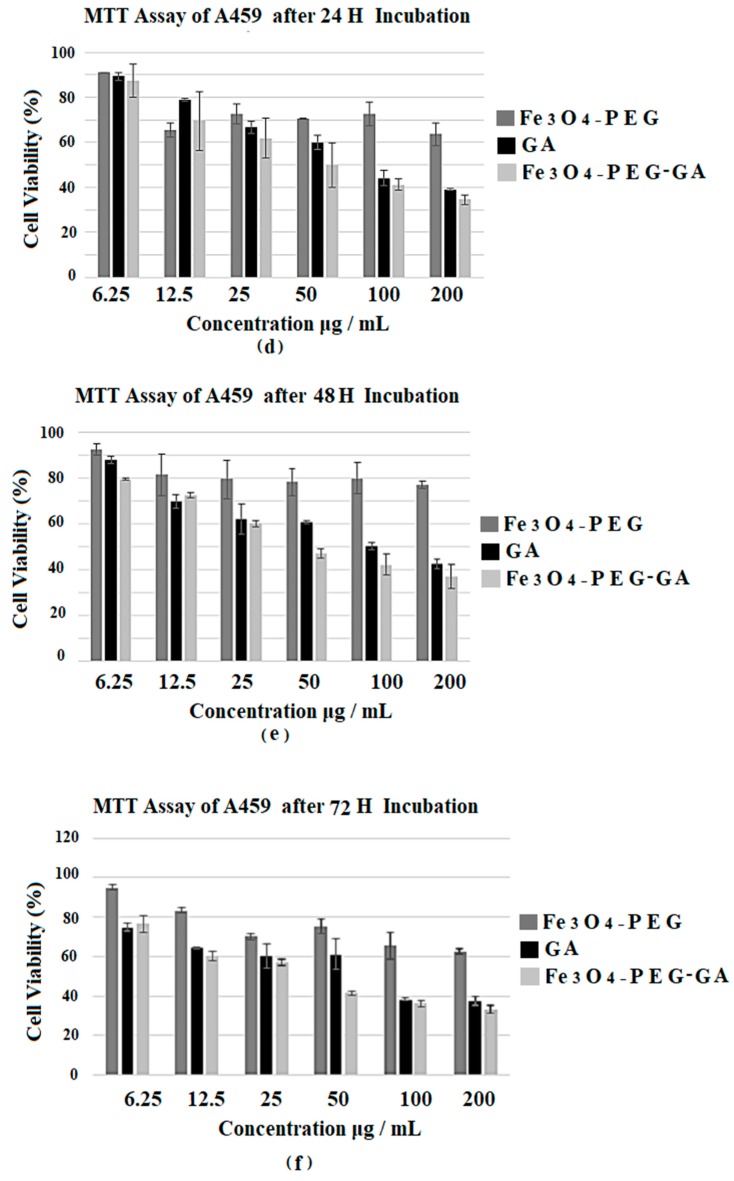

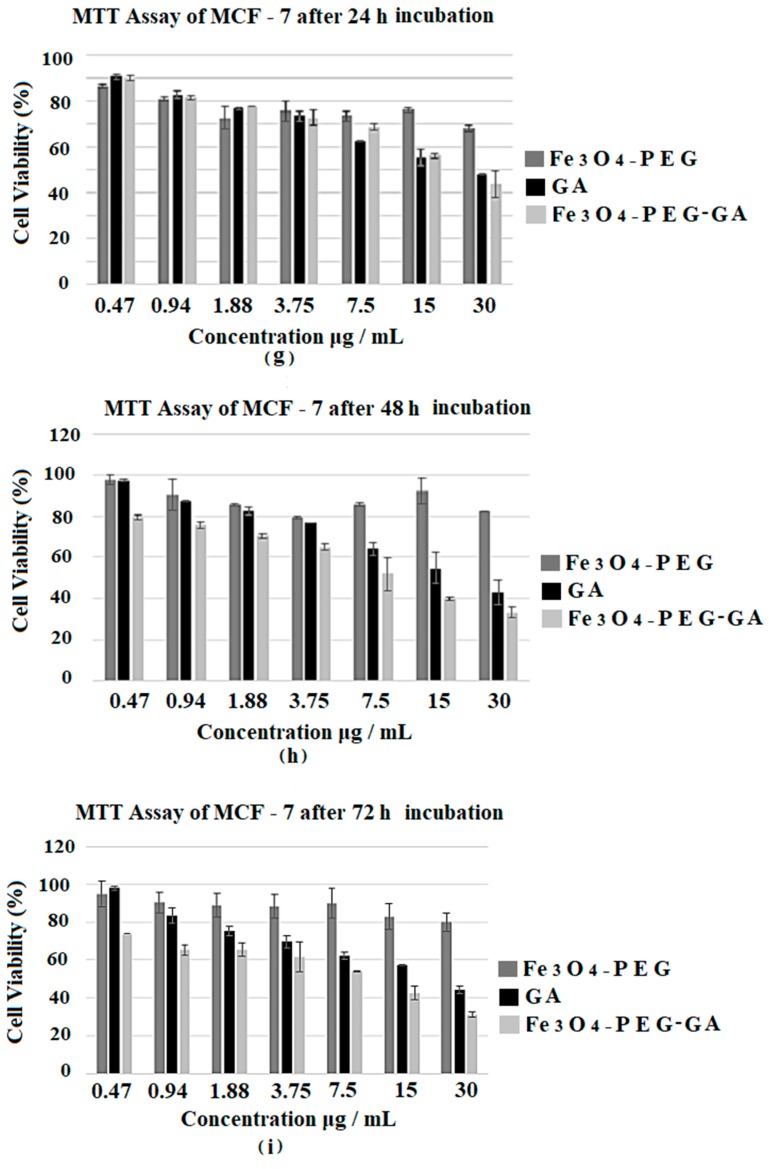

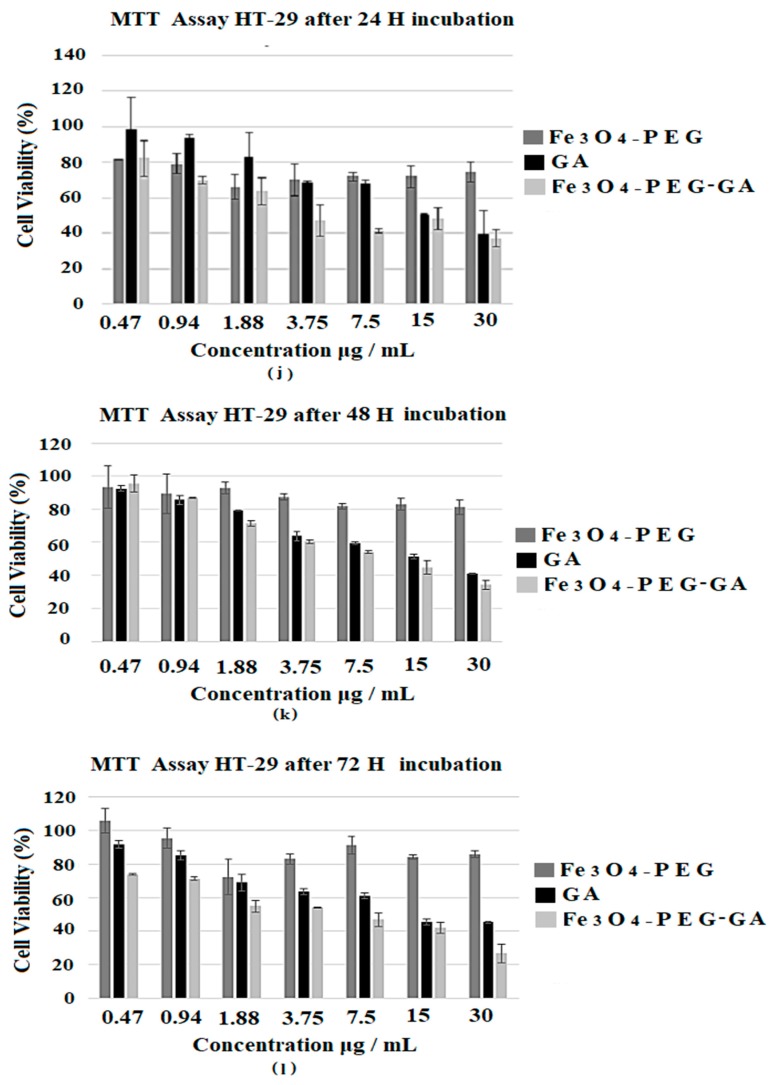
(**a**–**c**) shows the cell viability (%) of 3T3 cells estimated by MTT assay after 24, 48 and 72 h incubation respectively; (**d**–**f**) shows the cell viability (%) of A549 cells estimated by MTT assay after 24, 48 and 72 h incubation respectively; (**g**–**i**) shows the cell viability (%) of MCF-7 cells estimated by MTT assay after incubation for 24, 48 and 72 h respectively; (**j**–**l**) shows the cell viability (%) of HT-29 cells estimated by MTT assay after 24, 48 and 72 h incubation respectively.

All the samples e.g., designed anticancer nanocomposite (Fe_3_O_4_-PEG-GA), empty nanocarrier (Fe_3_O_4_-PEG) and free dug GA was tested against 3T3 fibroblast cells for cytotoxicity evaluation using their gradient concentrations i.e., (0.47, 0.94, 1.88, 3.75, 7.5, 15 and 30 µg/mL) and were incubation for 24, 48 and 72 h as shown in Figure 2a–c respectively. Cell viability studies revealed that free drug GA, empty carrier and the designed nanocomposite (Fe_3_O_4_-PEG-GA) were found to be biocompatible with 3T3 cell as the percentage cell viability was found to be about 80% even after 24, 48 and 72 h incubation at maximum concentration of 30 μg/mL as shown in Figure 2a–c. The MTT results showed that neither GA, Fe_3_O_4_-PEG nor Fe_3_O_4_-PEG-GA caused toxicity to 3T3 cells at all time points. As we previously reported nanocomposite (P-Fe_3_O_4_-PEG-GA) was also found to be biocompatible with MRC-5 human normal lungs cells with percentage cell viability of 100% after 72 h incubation [31]. These results indicate the high biocompatibility of all the samples.

#### 2.1.6. Anticancer Assays

The free drug GA, empty nanocarrier (Fe_3_O_4_-PEG) and designed magnetite nanocomposite formulation (Fe_3_O_4_-PEG-GA) were tested against different cancer cell line namely lung cancer cell (A549), breast cancer cell (MCF-7) and colon cancer cell (HT-29) lines to determine their anticancer properties. In our previous studies we had tested the nanocomposite (P-Fe_3_O_4_-PEG-GA) against breast cancer cell (MCF-7) in this study we repeated the assay on breast cancer cell (MCF-7) as the percentage drug loading of GA is different than previously reported (P-Fe_3_O_4_-PEG-GA). Doxorubicin, a chemotherapeutic drug, has been studied extensively and its IC_50_ values towards the same cancer cell lines have been reported to be 0.33 ± 0.03, 0.05 ± 0.01 and 0.58 ± 0.01 µg/mL for HT-29, MCF-7 and A549 respectively [33].These IC_50_ values of Doxorubicin were used as a reference for the positive control in this study.

#### 2.1.7. Anticancer Activity against Lung Cancer Cell (A549)

Figure 2d–f shows the percentage cell viability of A549 lung cancer cells treated with higher concentrations i.e., 6.25, 12.5, 25, 50, 100 and 200 μg/mL of the free drug GA, empty nanocarrier Fe_3_O_4_-PEG and the nanocomposite Fe_3_O_4_-PEG-GA. Previous anticancer studies involving A549 used a higher range of concentration of GA as well, ranging from a minimum of 10 µg/mL up to 500 µg/mL [34,35,36]. All the samples with above concentrations were incubated for 24, 48 and 72 h with the A549 lung cancer cells as shown in Figure 2d–f respectively. The designed anticancer nanocomposite Fe_3_O_4_-PEG-GA showed better anticancer activity (IC_50_ 37.49 µg/mL) compared to free drug GA (IC_50_ 56.49 µg/mL). The effective IC_50_ concentration (i.e., actual amount of GA based on percentage drug loading) present in the nanocomposite 13.12 µg/mL is much lower than IC_50_ of whole nanocomposite 37.4 µg/mL. So in reality, effective IC_50_ of nanocomposite 13.121 µg/mL compared to IC_50_ free GA (i.e., 56.49 µg/mL) much lower against lung cancer cells A549. Table 1 shows the IC_50_ of free drug GA, and the nanocomposite Fe_3_O_4_-PEG-GA compared to Doxorubicin.

#### 2.1.8. Anticancer Activity against Breast Cancer Cell MCF-7 Cells

All the samples i.e., free drug GA, empty nanocarrier (Fe_3_O_4_-PEG) and anticancer nanocomposite (Fe_3_O_4_-PEG-GA) were tested against breast cancer cell MCF-7 cells to screen their anticancer activity. Different gradient concentrations i.e., 0.47, 0.94, 1.88, 3.75, 7.5, 15 and 30 μg/mL of all the samples were incubated for 24, 48 and 72 h with breast cancer cell MCF-7 cells and results are shown in Figure 2g–i. A range of cytotoxicity in a time and dose-dependent manner was observed. The IC_50_ value of the nanocomposite Fe_3_O_4_-PEG-GA 7.28 μg/mL and its effective IC_50_ 2.548 μg/mL are much lower than free drug GA IC_50_ 21.35. The IC_50_ of the this redesigned nanocomposite Fe_3_O_4_-PEG-GA nanocomposite in this in vitro study against breast cancer cell MCF-7 cells is much lower than our previously reported nanocomposite P-Fe_3_O_4_-PEG-GA [31]. The better anticancer activity of nanocomposite can be attributed to the nanosize and sustained release properties of the anticancer nanocomposite. Table 1 shows the IC_50_ vales of the nanocomposite and free drug GA against Doxorubicin.

#### 2.1.9. Anticancer Activity against Colon Cancer Cell (HT-29)

The free drug GA, empty nanocarrier and the nanocomposite Fe_3_O_4_-PEG-GA were tested against colon cancer cell (HT-29) using different concentrations i.e., 0.47, 0.94, 1.88, 3.75, 7.5, 15 and 30 μg/mL and incubated for 24, 48 and 72 h as shown in Figure 2j–l respectively. The IC_50_ of free drug GA was found to be 15 μg/mL. The IC_50_ of the nanocomposite Fe_3_O_4_-PEG-GA was found 4.85 μg/mL and the effective IC_50_ of the nanocomposite Fe_3_O_4_-PEG-GA in 4.85 μg/mL was calculated to be 1.697 μg/mL based on 35% GA loading. The IC_50_ values are given in Table 1.

## 3. Discussion

In this study we have redesigned our previously reported an anticancer nanocomposite formulation of Gallic acid loaded on iron oxide magnetite nanoparticles coated with polyethylene glycol (Fe_3_O_4_-PEG-GA). The XRD patterns of the nanocomposite Fe_3_O_4_-PEG-GA showed the characteristic peaks of iron oxide magnetite nanoparticles (Fe_3_O_4_), PEG and of the pure drug peaks are present with slight lesser intensity. The presence of characteristic peaks of Fe_3_O_4_, PEG and GA in the final anticancer nanocomposite confirms the successful formation of the nanocomposite Fe_3_O_4_-PEG-GA. The average particle size of the anticancer nanocomposite was found to be 10 nm with a narrow size distribution of 5–12 nm compared to previously reported designed (P-Fe_3_O_4_-PEG-GA) which had wide distribution 20–50 nm with average size of 31.44 nm [31]. The Percentage GA loading is found to be 35% in this redesigned nanocomposite is much higher compared to our previously reported nanocomposite of (Fe_3_O_4_-PEG-GA) with 7%. In addition to this in vitro release of GA from the nanocomposite was found to be highly sustained which took about 200 h (about 8 days) in human body simulated phosphate buffer saline (PBS) solution of pH 7.4 (blood pH) and pH 4.8 (intracellular lysosomal pH) at human body temperature 37 °C. In this study we tested the free drug GA, empty nanocarrier and the anticancer nanocomposite formulation Fe_3_O_4_-PEG-GA against A549 human lung carcinoma cells, *HT29* human colon adenocarcinoma cell line, repeated on MCF-7 breast cancer cells and with normal 3T3 cells for incubation period of 24, 48 and 72 h. The IC_50_ of the designed nanocomposite Fe_3_O_4_-PEG-GA are found to be much lower compared to free drug GA. The effective IC_50_ which is based on percentage drug (GA) loading in nanocomposite Fe_3_O_4_-PEG-GA which is even further lower. Table 1 shows the details of the IC_50_ against A549 human lung carcinoma cells, *HT29* human colon adenocarcinoma cell line, and MCF-7 breast cancer cells and with normal 3T3 cells.

## 4. Materials and Methods

### 4.1. Chemicals

Gallic acid of 97% purity, iron oxide coated with polyethylene glycol (PEG) nanocarrier (Fe_3_O_4_-PEG) and gallic acid-iron oxide coated with PEG nanocomposite (Fe_3_O_4_-PEG-GA) were provided by the Material Synthesis and Characterization Laboratory, Institute of Advance Technology University Putra Malaysia (Serdang Selangor Malaysia). All three drugs were used for the preliminary screening of the effectiveness between gallic acid nanocomposite and pure gallic acid against normal cell and three different types of cancer cell lines. To prepare the stock solution, 5 mg of each drug was initially dissolved in 200 µL of dimethyl sulfoxide (DMSO) before the mixture was vortexed and sonicated for at least 30 min to ensure that the drug was completely dissolved. Upon sonication, 800 µL of RPMI 1640 (Nacalai Tesque, Kyoto, Japan) was added and then vortexed for 2 min to make the total volume of 1 mL. The drug sub stock was further diluted to a series of concentrations (0.47–200 µg/mL) and was used on the same day it was prepared. MTT [3-(4,5-dimethylthiazol-2-yl)-2,5-diphenyltetrazolium bromide] and Trypsin-EDTA (Ethylene diamine tetraacetate) (0.25%) were purchased from Nacalai Tesque (Kyoto, Japan).

### 4.2. Cell Lines

Normal human fibroblast cells (3T3), human lung cancer epithelial cells (A549), human breast cancer epithelial cells (MCF-7) and human colon cancer epithelial cells (HT-29) were obtained from the American Tissue Culture Collection (Manassas, VA, USA). The cells were maintained in Roswell Park Memorial Institute (RPMI) 1640 medium (Nacalai Tesque, Kyoto, Japan). The growth medium was supplemented with 10% fetal bovine serum, l-glutamine 15 mmol/L, penicillin 100 U/mL and streptomycin 100 µg/mL. All cells were incubated at 37 °C in humidified 5% CO_2_/95% air and the media was replaced every 2 to 3 days.

### 4.3. In Vitro Cell Viability Assay

The tetrazolium-based Colorimetric Assay (MTT) was used to determine the cytotoxicity of the drugs on the cancer and normal cell lines. In the case of viable cells in the in vitro MTT assay, the (nicotinamide adenine dinucleotide phosphate) NADPH-dependent oxidoreductase enzyme that is produced by the healthy mitochondria of a living cell will be secreted outside the cell membrane and converts the tetrazolium dye into a purple colored compound called formazan. Cells were first maintained in drug free media until the cells reached 80–90% confluency. MTT assay was initiated by the seeding process of plating 1 × 10^5^ cells into each well of a flat bottomed 96-well plate making the total volume of media in each well 100 µL. The cells were then left to attach for 24 h. Cells were then treated with increasing concentrations of pure gallic acid (GA), iron oxide-PEG nanocarrier (Fe_3_O_4_-PEG) and gallic acid-iron oxide coated with PEG nanocomposite (Fe_3_O_4_-PEG-GA) to make the final volume of 200 µL per well and incubated in 5% CO_2_ at 37 °C for 24, 48 and 72 h. At the desired time point, 20 µL of MTT solution (5 mg/mL in PBS) was added to each well and kept in the incubator for 4 h before the cells were centrifuged to discard the supernatant. 100 µL of DMSO was later added and left in the dark for 30 min to dissolve the crystal formazan. Absorbance was measured at 570 and 630 nm was used to measure the background absorbance. Experiments were done in triplicates and cell viability was calculated based on the given formula. 

$$\mathrm{Cell}\;\mathrm{Viability}\;\left(\%\right)=\left\{\;\left({\mathrm{OD}}^{*}\;\mathrm{of}\;\mathrm{Treated}\right)÷\;\left({\;\mathrm{OD}}^{*}\;\mathrm{of}\;\mathrm{Control}\right)\times 100\right\}\;{\mathrm{OD}}^{*}=\mathrm{Optical}\;\mathrm{density}$$

### 4.4. Preparation of Magnetite Nanoparticles (Fe_3_O_4_), Polyethylene Glycol (PEG) Coating and GA Loading

Iron oxide magnetite nanoparticles (Fe_3_O_4_) were prepared by previously reported method, in brief in 80 mL deionized water containing 6 mL ammonia hydroxide (25%) a mixture of 2.43 g (FeCl_2_⋅4H_2_O) and 0.99 g (FeCl_3_⋅6H_2_O) was added. After adding the above material sample was subject to ultrasonication for 1 h. Sample was centrifuged and washed thoroughly with water and dried in oven at 80 °C for 24 h and ground to fine powder [15,26,31]. PEG coating was carried out by dissolving the 0.2 g of dried iron oxide magnetite nanoparticles (Fe_3_O_4_) in 1% of PEG solution followed by stirring for one hour after that sample was centrifuged and washed with ethanol thoroughly. GA was loaded on the designed nanocarrier Fe_3_O_4_-PEG by putting this sample to 50 mL (40 mL methanol and 10 mL water) of 1% GA and stirred for 24 h resulting in the formation of Fe_3_O_4_-PEG-GA. Next day sample was washed thoroughly with methanol and water by centrifugation. After that sample was dried in oven and ground to powder and subjected to further characterization.

## 5. Conclusions

The designed magnetite nanocomposite Fe_3_O_4_-PEG-GA was found to have higher drug GA loading 35% compared to previously reported nanocomposite with 7% GA loading. The in vitro release in human physiological pH 7.4 and pH 4.8 at 37 °C was conducted and was found to be highly sustained for up to 8 days compared to previously reported nanocomposite which took 3.5 days for the complete release and that was conducted at room temperature. In this study the magnetite nanocomposite Fe_3_O_4_-PEG-GA was found to be biocompatible with normal 3T3 cells. Both GA and Fe_3_O_4_-PEG-GA nanocomposite displayed time and dose-dependent anticancer activity against A549, MCF-7 and HT-29 cells Most importantly the IC_50_ of the magnetite nanocomposite Fe_3_O_4_-PEG-GA were found against these cell lines were much better than free drug GA. These in vitro study results are highly encouraging to go further animal studies.

## Figures and Tables

**Table 1:** The IC_50_ values of GA, Doxorubicin and Fe_3_O_4_-PEG-GA on cancer cell lines.

Cancer Cell Lines	IC_50_ (μg/mL) *			Effective IC_50_ (μg/mL) **
	GA	Fe_3_O_4_-PEG-GA	Doxorubicin	Fe_3_O_4_-PEG-GA
HT29	14.52 ± 0.94	4.85 ± 0.33	0.33 ± 0.03	1.70
MCF-7	21.35 ± 4.14	7.28 ± 0.64	0.05 ± 0.01	2.55
A549	56.49 ± 4.31	37.49 ± 1.42	0.58 ± 0.01	13.1

* Values are expressed as the mean ± standard deviation of 3 replicates. The IC_50_ value is defined as the concentration of drug needed for 50% cell inhibition; ** Values of actual IC_50_ that were calculated based on 35% GA loading in the nanocomposite.
